# Tyrosine metabolism disorder and the potential capability of anaerobic microbiota to decrease the value of aromatic metabolites in critically ill patients

**DOI:** 10.1186/cc14063

**Published:** 2014-12-03

**Authors:** N Beloborodova, V Moroz, A Osipov, A Bedova, Y Sarshor, A Vlasenko, A Olenin

**Affiliations:** 1 Negovsky Research Institute of General Reanimatology, Russian Academy of Medical Science, Moscow, Russia

## Introduction

**Figure 1:**
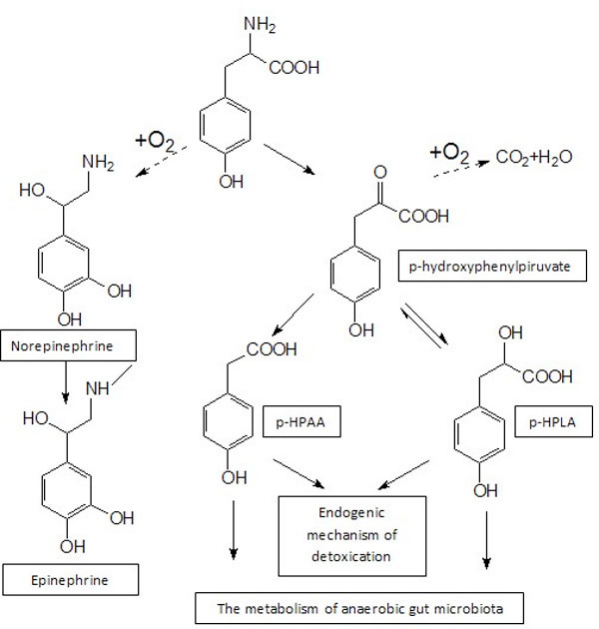


Metabolism of tyrosine can be switched in conditions of hemodynamic instability and tissue hypoperfusion. The violation of the oxygen-dependent metabolism of tyrosine must be accompanied by the activation of alternative pathways (Figure 1). Previously, we found a high level of content in the blood of aromatic intermediates p-hydroxyphenyllactic acid (p-HPLA) and p-hydroxyphenylacetic acid (p-HPAA) in patients with sepsis [1,2]. We assume that the anaerobic microbiota can take an important part in biodegradation of excess alternative metabolites of aromatic amino acids [3,4].

## Methods

**Figure 2:**
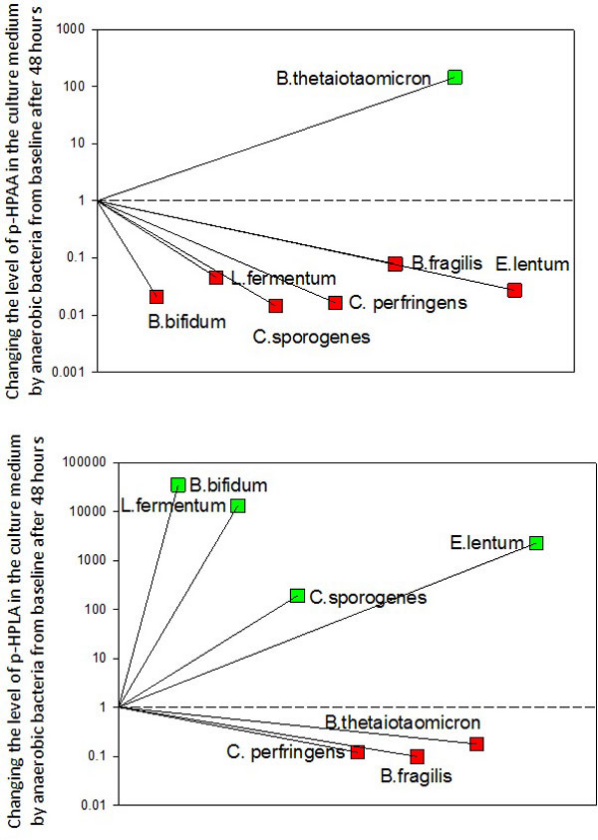


Serum samples were collected from critically ill patients (*n *= 65) with surgical diseases (*n *= 32), brain injury (*n *= 22), and lung diseases (*n *= 11). Patients were included in the study on the day of admission to the ICU. The median of age was 62 (IR 42 to 77) years, the APACHE II score was 17 (IR 11 to 29). The level of p-HPLA and p-HPAA were measured in serum using GC-FID. Anaerobic bacteria (Figure 2) were cultured in Shedler media, and the level of aromatic metabolites were measured before and after 48 hours of incubation using GC-MS. Data were compared by Mann-Whitney *U *test, *P *< 0.05 considered significant (IBM SPSS Statistics 22).

## Results

In surviving patients (*n *= 24) the total level of p-HPLA and p-HPAA (4.47; 3.24 to 8.35 µM) was less (*P *< 0,001) than in patients who died (*n *= 41) (13.67; 5.78 to 52.26 µM). The severity of organ dysfunction on a SOFA scale correlates (*r*_s _= 0.7, *P *< 0.001) with the total level of the p-HPLA and p-HPAA. Also the total level of aromatic compounds correlates with lactate (*r*_s _= 0.6; *P *< 0.001), BE (*r*_s _= -0.5, *P *< 0.001) and perfusion blood pressure (*r*_s _= -0.5, *P *< 0.001). ROC analysis revealed that p-HPLA has the largest area under the curve (0.78; CI 0.67 to 0.90, *P *< 0.001). In experimental studies, anaerobic bacteria significantly reduced the level of p-HPAA and p-HPLA (Figure 2).

## Conclusion

High level of p-HPLA and p-HPAA correlate with severity and mortality of patients. Hypoxia can be one of the leading mechanisms of tyrosine metabolism disorders in critically ill patients. *Bacteroides *spp. are able to consume p-HPLA and p-HPAA and consequently may be involved in the elimination of these intermediates from the human body mutually with endogenous mechanisms of detoxification.
